# Therapeutic Effectiveness and Safety of Repurposing Drugs for the Treatment of COVID-19: Position Standing in 2021

**DOI:** 10.3389/fphar.2021.659577

**Published:** 2021-06-14

**Authors:** Safaet Alam, Taslima Binte Kamal, Md. Moklesur Rahman Sarker, Jin-Rong Zhou, S. M. Abdur Rahman, Isa Naina Mohamed

**Affiliations:** ^1^ Department of Pharmacy, State University of Bangladesh, Dhaka, Bangladesh; ^2^ Pharmacology and Toxicology Research Division, Health Med Science Research Limited, Dhaka, Bangladesh; ^3^ Nutrition/Metabolism Laboratory, Beth Israel Deaconess Medical Center, Harvard Medical School, Boston, MA, United States; ^4^ Department of Clinical Pharmacy and Pharmacology, Faculty of Pharmacy, University of Dhaka, Dhaka, Bangladesh; ^5^ Pharmacology Department, Medical Faculty, Universiti Kebangsaan Malaysia (The National University of Malaysia), Kuala Lumpur, Malaysia

**Keywords:** COVID-19, repurposing drugs, favipiravir, remdesivir, doxycycline, hydroxychloroquine, ivermectin, rivaroxaban

## Abstract

COVID-19, transmitted by SARS-CoV-2, is one of the most serious pandemic situations in the history of mankind, and has already infected a huge population across the globe. This horrendously contagious viral outbreak was first identified in China and within a very short time it affected the world's health, transport, economic, and academic sectors. Despite the recent approval of a few anti-COVID-19 vaccines, their unavailability and insufficiency along with the lack of other potential therapeutic options are continuing to worsen the situation, with valuable lives continuing to be lost. In this situation, researchers across the globe are focusing on repurposing prospective drugs and prophylaxis such as favipiravir, remdesivir, chloroquine, hydroxychloroquine, ivermectin, lopinavir-ritonavir, azithromycin, doxycycline, ACEIs/ARBs, rivaroxaban, and protease inhibitors, which were preliminarily based on *in vitro* and *in vivo* pharmacological and toxicological study reports followed by clinical applications. Based on available preliminary data derived from limited clinical trials, the US National Institute of Health (NIH) and USFDA also recommended a few drugs to be repurposed i.e., hydroxychloroquine, remdesivir, and favipiravir. However, World Health Organization later recommended against the use of chloroquine, hydroxychloroquine, remdesivir, and lopinavir/ritonavir in the treatment of COVID-19 infections. Combining basic knowledge of viral pathogenesis and pharmacodynamics of drug molecules as well as *in silico* approaches, many drug candidates have been investigated in clinical trials, some of which have been proven to be partially effective against COVID-19, and many of the other drugs are currently under extensive screening. The repurposing of prospective drug candidates from different stages of evaluation can be a handy wellspring in COVID-19 management and treatment along with approved anti-COVID-19 vaccines. This review article combined the information from completed clinical trials, case series, cohort studies, meta-analyses, and retrospective studies to focus on the current status of repurposing drugs in 2021.

## Introduction

Coronaviruses, genus of the Coronaviridae family, have been the most tragic pathogen of the 21st century, causing three major epidemics since 2002. The recent coronavirus outbreak of 2019 is the most dreadful of all the previous ones, halting every aspect of human life worldwide. The World Health Organization (WHO) recognized coronavirus disease-2019 (COVID-19) as a pandemic on March 11, 2020 (Singh et al., 2020). According to WHO, a novel coronavirus (severe acute respiratory syndrome coronavirus 2, SARS-CoV-2) case, found in China, was first reported to the WHO Country Office on December 31, 2019. Up to January 25, 2021, 98,794,942 confirmed cases and 2,124,193 deaths had been reported globally due to COVID-19 (WHO Coronavirus Disease (COVID-19) Dashboard, 2020). SARS-CoV- 2 contains a receptor binding domain on its surface’s crown shaped spikes protein which helps it to bind to human angiotensin-converting enzyme 2 (ACE2), promoting its endocytosis into human lung cells. After entry, this virus continues its replication in lung cells by synthesizing and assembling its own protein using host cells’ protein synthesis machinery (Rothan and Byrareddy, 2020).

Although a few vaccines against SARS-CoV-2 infections have recently been approved by the regulatory authorities and been used, there are no other proven and/or potential therapeutic options in hand to treat this pandemic. To worsen the issue, allocation and production in sufficient quantities and the affordability and deployment of these vaccines are also notable challenges faced by the policy makers and developers (Folegatti et al., 2020; Polack et al., 2020; Logunov et al., 2021; Wouters et al., 2021). Thus, as the search for new effective therapeutic options are still being conducted and will need more time and resources, scientists are exploring the possibility of the usage of existing drugs that may be therapeutically effective for the treatment of COVID-19 along with the discovery and availability of vaccines. Repurposing of a drug indicates the use of currently existing drugs for new therapeutic purposes rather than its primary and main indications (Khan et al., 2020). There have been several investigations on drug repurposing for COVID-19 recently and clinical trials are still being conducted on several drugs. The current review aims to present a clear idea on the mechanism of actions and outcomes from completed clinical trials along with the data from case series, retrospective studies, cohort studies, and meta-analyses on the efficacy and safety of prospective repurposing drugs in the treatment of COVID-19 infections.

### Article Search Strategy

To summarize and compile the desired findings related to repurposing drugs in COVID-19 treatment, a literature search was conducted using Web of Science, PubMed, ScienceDirect, Scopus, Wiley Online Library, and Google Scholar databases. In addition, clinical trial reports and other relevant information were also checked critically from authentic sources including WHO, USFDA, and ClinicalTrials.gov websites. The keywords used during the searches included ‘COVID-19’, ‘COVID’, ‘Repurposing drugs’, ‘Anti-COVID-19 drugs’, ‘Coronavirus’ and ‘Clinical trial’, ‘Case study’, ‘Retrospective study’, ‘Cohort study’, and ‘Meta-analysis’. Only the completed studies on clinical trials, case series, cohort studies, meta-analyses and retrospective data (up to 9th April, 2021) from aforementioned authentic databases were included in this review, while incomplete studies of any of the above categories were excluded. Of the 412 identified papers and reports, after discarding titles and abstracts mismatched with the inclusion criteria, 176 unique sources were identified to be finally included in the review.

### Remdesivir

Remdesivir, developed by Gilead Sciences, was considered as a potential treatment option for RNA viruses like EBOV (Ebola Virus), MERS (Middle East respiratory syndrome), and SARS (Severe acute respiratory syndrome), all of which could cause a global pandemic. In 2014, during the ebola virus outbreak, clinical evaluation for remdesivir started. But being inferior to antibody-based therapeutics for the treatment of EBOV and having one serious adverse event (hypotension) along with increased plasma levels of creatinine and aspartate aminotransferase, its evaluation study against Ebola virus was terminated (Eastman et al., 2020). A randomized controlled trial of four investigational therapies (triple monoclonal antibody ZMapp, remdesivir, the single monoclonal antibody MAb114, or the triple monoclonal antibody REGN-EB3) was conducted for ebola virus disease (EVD) after the EVD outbreak of 2018 in the Democratic Republic of Congo. Data from this trial indicated that remdesivir was inferior to the other therapies and the highest mortality was reported in the remdesivir group than the other groups. Random assignment of patients to the remdesivir group was recommended to be stopped depending on efficacy and mortality analysis of patients. The median time of the first negative result for EBOV nucleoprotein was also higher in the remdesivir group (28 days) compared to the other groups (Mulangu et al., 2019). With the COVID-19 outbreak, several *in-vitro* and *in-vivo* animal studies were conducted to assess the effectiveness of remdesivir against SARS-CoV-2 and most of the studies showed positive results. A recent *in vitro* study in infected Vero E6 involving qRT-PCR quantification of viral copy number demonstrated an IC50 of 770 nM (Wang et al., 2020).

Supportive *in-vitro* data for the use of remdesivir against SARS-CoV-2 and a lack of alternative treatment options led to the development of clinical trials with remdesivir. While clinical trials with remdesivir were being developed in China, the first case of a COVID-19 positive patient was reported in the USA on January 20, 2020. The patient was given IV remdesivir infusion and showed improved clinical condition with no identified adverse event. However, the patient also received Paracetamol, Ibuprofen, Guaifenesin, Vancomycin, and Cefepime along with remdesivir (Holshue et al., 2020). A benefit risk assessment study for remdesivir showed that one clinical trial reported non-significant benefit of recovery time (21 vs. 23 days) while another study reported non-significant reduction of mortality risk (8 vs. 12%) compared to placebo (Davies et al., 2020).

**FIGURE 1:**
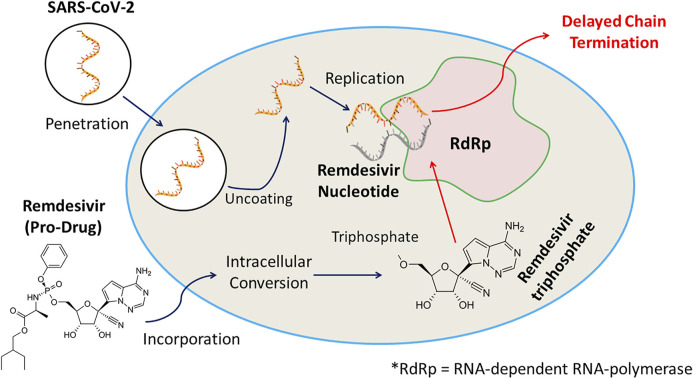
Remdesivir, an investigational nucleotide analogue, and its triphosphate possessing resemblance with Adenosine triphosphate can compete with it to act as a substrate for various viral RNA polymerase inhibiting RNA synthesis. Remdesivir triphosphate form adds to the growing RNA chain more efficiently than ATP, causing chain termination at 3 nucleotides downstream, and displays anti-COVID-19 efficacy.

Remdesivir is an investigational nucleotide analogue and as its triphosphate form resembles Adenosine triphosphate it can compete with it to act as a substrate for various viral RNA polymerase inhibiting RNA synthesis. Remdesivir triphosphate form adds to the growing RNA chain more efficiently than ATP, causing chain termination at 3 nucleotides downstream. This delayed chain termination mechanism can contribute to the effectiveness of remdesivir against all strain of coronaviruses including SARS-CoV and SARS-CoV-2 (Saha et al., 2020). The anti-COVID-19 action of remdesivir is displayed in Figure 1.

Remdesivir, being an investigational drug, received the first FDA authorization for emergency use in treating SARS-CoV-2 only in hospital or healthcare settings capable of providing acute care. Further research is required to establish its role in managing COVID-19 patients (Ridwan, 2020).

A retrospective cohort study in Korea involving 86 severe COVID-19 patients hospitalized between June 1 and July 31, 2020, suggested better clinical and virologic effectiveness of remdesivir compared to supportive care in the case of mechanical ventilator requirement (22.9 vs. 44.7%, *p* = 0.032), mechanical ventilator duration (average, 1.97 vs. 5.37 days; *p* = 0.017), and viral load reduction (increase of cycle threshold (Ct) values: average, 10.19 vs. 5.36; *p* = 0.007 and the slope of Ct value increase: average, 5.10 vs. 2.68; *p* = 0.007) (Joo et al., 2021). Another retrospective analysis demonstrated a well-tolerated safety profile of remdesivir and 84% improvement rate of the drug in COVID-19 patients (Gupte et al., 2021). On the contrary, a large observational study in Japan concluded that remdesivir may not have any benefit on hospitalized COVID-19 patients’ clinical outcomes and reductions in need of invasive/non-invasive respiratory support. Results from the study showed that the remdesivir case group and non-remdesivir control group had no significant difference in fatality risk (12.2 vs. 13.3%; *p* = 1.0), risk of invasive mechanical ventilation or extracorporeal membrane oxygenation (IMV/ECMO 5.4 vs. 4.6%; *p* = 0.757), length of intensive care unit stay (6 vs. 6 days; *p* = 0.473), or length of IMV (13 vs. 5 days; *p* = 0.509), while longer length of stay (LoS) in healthcare facilities was observed in the case group than in the control group (14 vs. 11 days; *p* < 0.001) (Tsuzuki et al., 2021). Again, a retrospective, cohort study involving 142 COVID-19 patients admitted to a large tertiary center in Israel showed no nasopharyngeal viral load changes with remdesivir (Goldberg et al., 2021).

The Adaptive COVID-19 Treatment Trial (ACTT-1) involving 1,062 patients and Solidarity involving 6,838 patients are the two largest trials among all the published randomized controlled trials (RCTs) aiming to evaluate remdesivir as a COVID-19 treatment option. ACTT-1 found reduced recovery time (shortened from 15 to 10 days) but no change in mortality (hazard ratio 0.73, 95% confidence interval [CI] 0.52–1.03) with remdesivir and Solidarity also showed no mortality benefit for the drug (rate ratio 0.95, 95% CI 0.81–1.11). Both the trials demonstrated a lack of effectiveness of remdesivir in critically ill patients (Wu and Morris, 2021). A meta-analysis of four randomized controlled trials (RCTs) of remdesivir in moderate or severe COVID-19 patients showed increased recovery rate and hospital discharge with no effect on clinical improvement and mortality (Al-Abdouh et al., 2021). Another meta-analysis involving five RCT’s concluded that remdesivir may elevate the recovery percentage and slightly decrease ventilation requirement in hospitalized COVID-19 patients but has no effect in mortality. It also suggested the benefit of a 5-days course over a 10-days course of remdesivir (Kaka et al., 2021). Six published case-series and cohort series studies on the use of remdesivir against COVID-19 reported potential effectiveness of the drug but most of the study results are limited by their small group size, short follow-up duration, lack of potential data, scarcity of patient’s information treated at baseline, and lack of a randomized control group (Saqrane et al., 2021).

Moreover, a few more studies are going on to determine the safety, antiviral efficacy, pharmacokinetic property, and tolerability of remdesivir alone and in combination with other agents like tocilizumab, merimepodib, and convalescent plasma therapy in cases of severe and moderate COVID-19 patients in the United States, Bangladesh, Nepal, China, and Canada (Anonymous, 2020a). However, with a lack of adequate evidence supporting remdesivir use in COVID-19, WHO recently deprecated its use regardless of hospitalized patients’ severity of illness (WHO, 2020).

### Doxycycline

Doxycycline (1967) is a semisynthetic second-generation class of tetracyclines with a broad spectrum of antimicrobial activity. It was developed by chemical modification of basic chlortetracycline structure and identified and named by mycologist B. M. Duggar in 1944 (Joshi and Miller, 1997). Having antiviral, immunomodulatory, and anti-inflammatory properties, doxycycline and other tetracycline antibiotics could be potential options for treating COVID-19 patients. An *in vitro* antiviral activity study of doxycycline involving VERO E6 cell line showed positive results against SARS-CoV-2 with median effective concentration (EC50) of 5.6 ± 0.4 µM. For prophylaxis, doxycycline could be used in combination with hydroxychloroquine or chloroquine (Gendrot et al., 2020).

**FIGURE 2:**
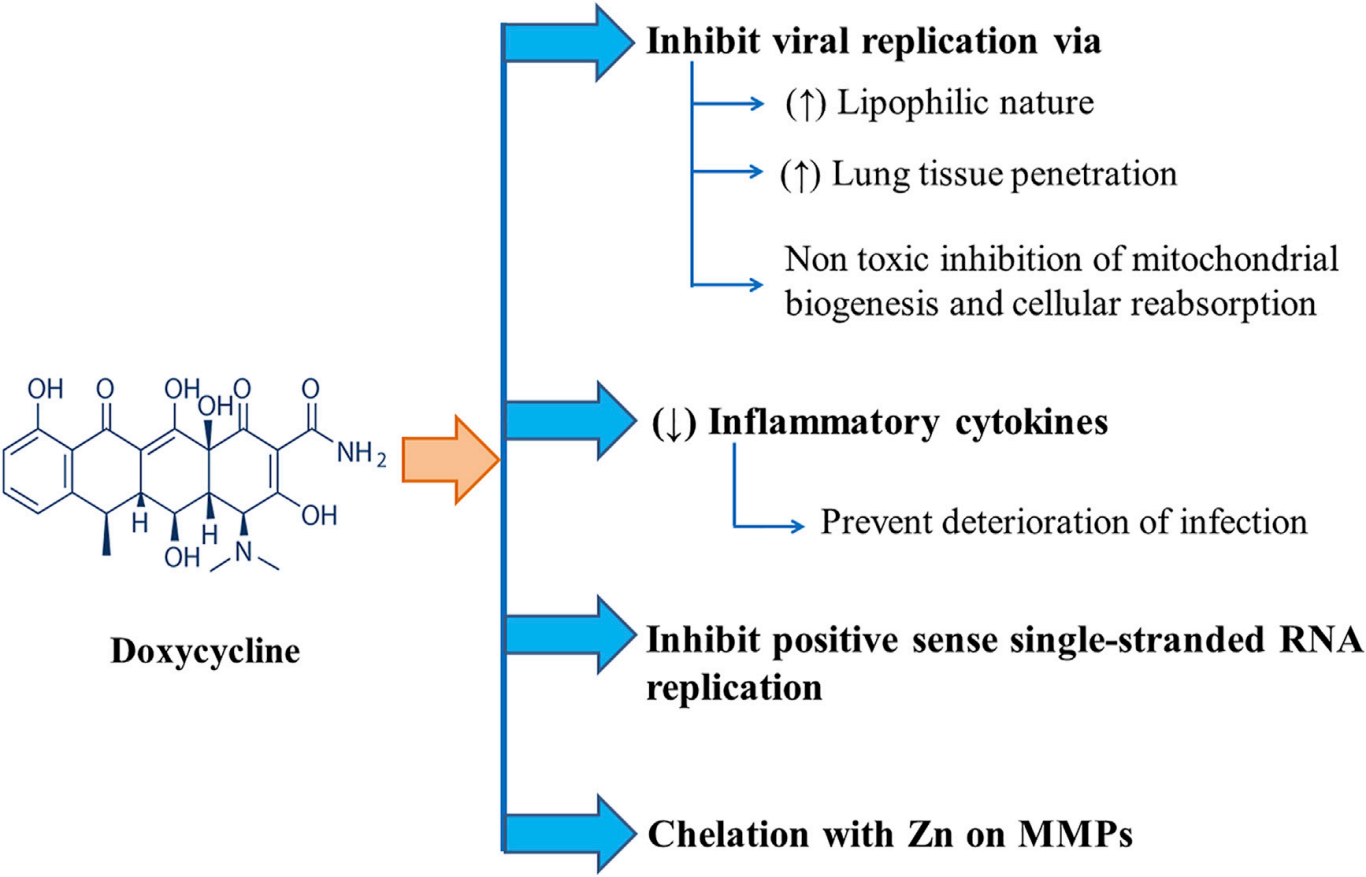
Different modes of Anti-COVID-19 actions of Doxycycline including inhibition of viral replication and positive sense single-stranded RNA replication along with chelation with Zn on MMPs and downregulation of inflammatory cytokines.

Several mechanisms have been proposed for the actions of doxycycline against SARS-CoV-2. Effectiveness of tetracyclines (e.g., tetracycline, doxycycline, and minocycline) against COVID-19 infection can be attributed to their higher lipophilicity with higher lung tissue penetration ability facilitating their role in viral replication inhibition in the lungs. Again, tetracyclines can form chelate with zinc compounds on matrix metalloproteinases (MMPs) of the host while coronaviruses rely heavily on MMPs of the host for cell adhesion, infiltration, survival, and replication, thus inhibiting the virus’ ability to replicate within the host. They might also act by inhibiting positive-sense single-stranded RNA replication of COVID-19. Again, due to their anti-inflammatory mechanism, tetracyclines are used to treat different inflammatory conditions. They can decrease the levels of inflammatory cytokines, such as tumor necrosis factor-a, interleukin (IL)-1b, and IL-6, which are elevated when lung tissue is infected with severe acute respiratory syndrome–associated coronavirus (SARS-CoV) deteriorating the infection itself (Sodhi and Etminan, 2020). The anti-COVID-19 action of doxycycline is displayed in Figure 2.

Doxycycline (a tetracycline antibiotic), being a non-toxic mitochondrial biogenesis and cellular respiration inhibitor and having various pleiotropic features, can attenuate the replication of most viruses which need the energy of aerobic glycolysis to replicate and use mitochondria as cellular powerhouses. It is suggested that doxycycline in combination with Vitamin C shows more effectiveness against COVID-19 (Szolnoky, 2020). Again, Sargiacomo et al. suggested that senolytic drugs such as doxycycline can be used for SARS-CoV-2 infection to prevent fibrotic transformation. SARS-CoV-2 can cause stormy inflammation and subsequent fibrosis after binding and replicating preferably with senescent and chronologically aged lung cells (Sargiacomo et al., 2020).

In New York, 89 high-risk COVID-19 patients (diagnosed from March 18 to May 13, 2020) received early treatment with doxycycline in long-term care facilities and demonstrated early clinical recovery, reduced hospitalization, and reduced mortality. Among 89 patients, 76 (85%) were clinically recovered, three patients (3%) showed deterioration of clinical condition requiring transfer to hospital, and 10 patients (11%) died (Alam et al., 2020). A prospective case-series study involving 21 COVID-19 patients from Iran from September 14, 2020 to September 28, 2020 suggested the potential effectiveness of doxycycline in COVID-19 treatment (Meybody et al., 2021). Recovery of four high risk COVID-19 patients with comorbid pulmonary diseases who were treated with doxycycline was reported (Narendrakumar et al., 2021).

Doxycycline shows more effectiveness against SARS-CoV-2 when used in combination with other drugs like hydroxychloroquine, ivermectin, and dapsone. More studies are required to establish its effectiveness in proposed combination therapies for COVID-19 (Farouk and Salman, 2020; Malek et al., 2020). At present, various clinical trials are being conducted with doxycycline alone or in combination with other drugs for COVID-19 treatment. An experimental trial with combination of hydroxychloroquine and doxycycline involving 54 high risk COVID-19 patients demonstrated higher clinical recovery, decreased hospital stay, and reduced mortality (Narendrakumar et al., 2021). A recent double-blind and randomized interventional trial of doxycycline in combination with ivermectin was conducted with 400 participants. Early clinical improvement was reported in 60.7% of patients (111 out of 183 patients) with doxycycline and ivermectin combination compared to 44.4% (80 out of 180) with placebo (NCT04523831, 2020). More studies are still in progress in Iraq, Egypt, and Bangladesh to assess the efficacy and safety of this combination for COVID-19 treatment (Anonymous, 2020b).

### Azithromycin

Azithromycin, a macrolide derivative and the first of the fifteen-membered ring azalide class of antimicrobials, shows a broad spectrum of activity against various gram-positive and gram-negative bacteria and other atypical pathogens (Ballow and Amsden, 1992). They act by inhibiting bacterial growth through interference with their protein synthesis. Azithromycin showed *in-vitro* activity against Ebola and Zika viruses and prevented severe viral infections of the respiratory tract. Previously they have been used as an adjunctive therapy to treat viral respiratory tract infections due to their anti-inflammatory and immunomodulatory activities in addition to extended antibacterial coverage (Wu et al., 2020). With no available definite treatment option for COVID-19, azithromycin has undergone several studies recently to find its effectiveness against SARS-CoV-2. An *in-vitro* screening with VeroE6 (ATCC CRL-1586) and Caco-2 (ATCC HTB-37) cells reveals Azithromycin to be a potential inhibitor of SARS-CoV-2 replication with EC_50_ (50% effective concentration) = 2.12 µM and EC_90_ (90% effective concentration) = 8.65 µM. (Touret et al., 2020).

**FIGURE 3:**
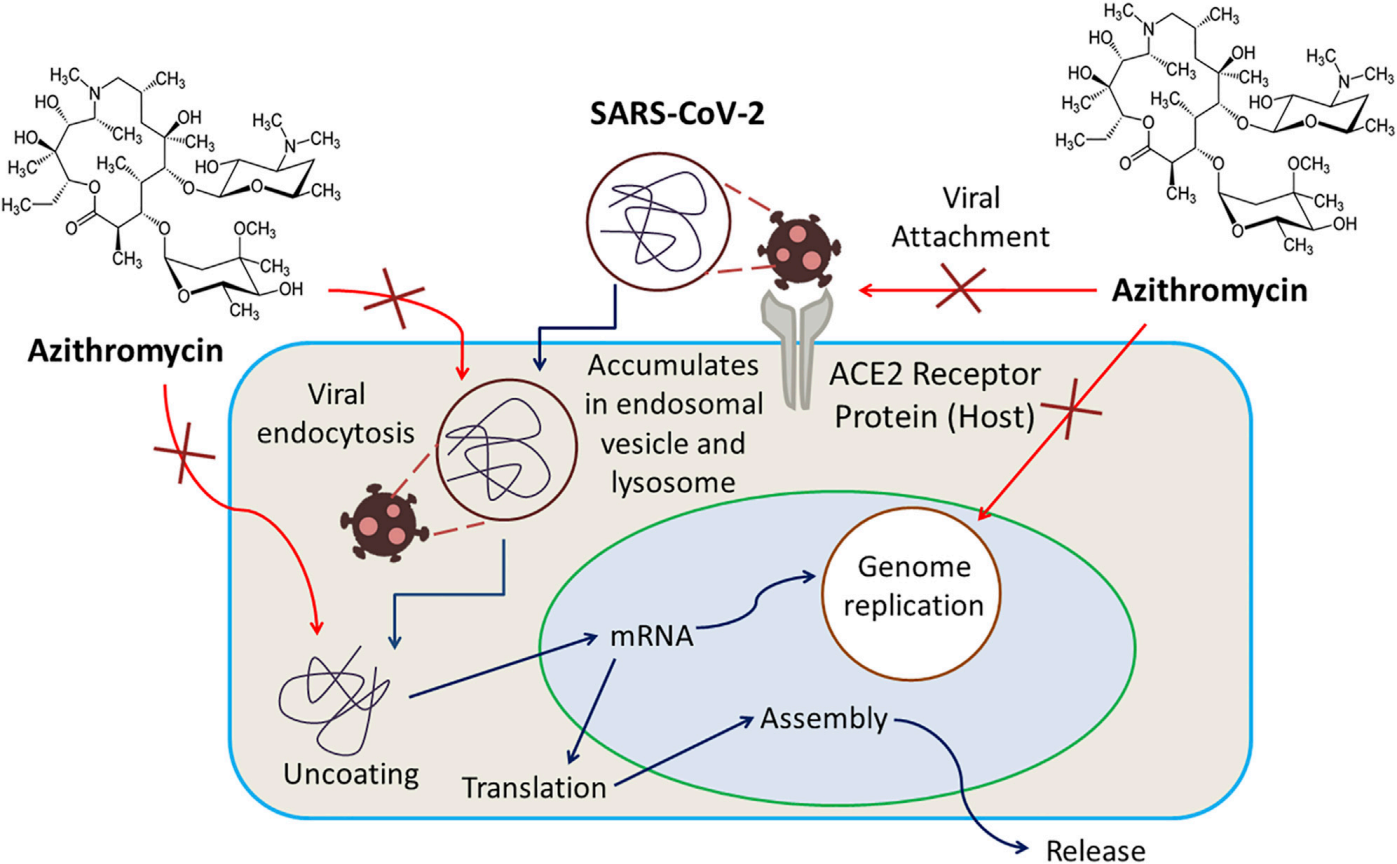
Azithromycin exerts inhibition of uncoating of coronaviruses, amplification of host’s interferon (IFN) pathway-mediated antiviral responses resulting in a reduction of viral replication, interference in the binding interaction of SARS-CoV-2 spike protein and host receptor ACE2 protein as well as viral endocytosis.

There is no known specific mechanism; instead, several mechanisms have been proposed for the probable antiviral activity of Azithromycin. Any of these mechanisms could be responsible for its activity against SARS-CoV-2. For example, being a weak base, azithromycin can inhibit the uncoating of coronaviruses as the uncoating of enveloped viruses requires an acidic environment. Again, preferential accumulation of this drug in lysosomes and endosomal vesicles can elevate pH and limit replication of the virus by blocking endocytosis. Another possible mechanism can be the amplification of host’s interferon (IFN) pathway-mediated antiviral responses, resulting in a reduction of viral replication. A quantum mechanical model proposes that azithromycin’s interference in the binding interaction of SARS-CoV-2 spike protein and host receptor ACE2 (angiotensin converting enzyme-2) protein can be responsible for limited viral entry (Damle et al., 2020). Azithromycin has also been suggested to have senolytic activity used to inhibit viral replication and IL-6 production (Sargiacomo et al., 2020). The anti-COVID-19 action of Azithromycin is displayed in Figure 3.

Currently, various clinical trials are being conducted to evaluate the efficacy of Azithromycin in combination with hydroxychloroquine for COVID-19 treatment. According to a study, azithromycin and hydroxychloroquine combination therapy was encouraged in the treatment of early symptomatic and high-risk outpatients (Esper et al., 2020). Several other preliminary trials also reported positive results like the one performed by Pfizer in France and another reported by Gautret et al. (2021) which previously suggested the use of combination treatment of azithromycin and hydroxychloroquine for COVID-19 (Gautret et al., 2021; Wu et al., 2020). According to another retrospective observational study, azithromycin (introduced after 6–8 h from being diagnosed) could notably improve the hospital stay duration and requirement of respiratory support in hospital days (Ishaqui et al., 2020). Despite having no clear idea about the optimal dose of azithromycin against SARS-CoV-2, in accordance with IDSA guidelines and RECOVERY trial, 500 mg azithromycin once daily (OD) for 5 days is recommended in the case of severe patients. Again, based on previous studies, azithromycin was only suggested to early phase patients with COVID-19 infection as in later phases it failed to deliver any beneficial impacts on clinical status (Echeverría-Esnal et al., 2021).

On the contrary, one of the biggest randomized controlled clinical trials conducted on hospitalized patients with mild to moderate COVID-19 infection involved the administration of hydroxychloroquine monotherapy azithromycin and hydroxychloroquine cotherapy, which did not alleviate patient status at 15 days compared to standard care (Arshad et al., 2020; Echeverría-Esnal et al., 2021). Another experiment also failed to demonstrate any evidence of rapid antiviral activity in severe COVID-19 patients with hydroxychloroquine and azithromycin combination therapy (Molina et al., 2020). Moreover, this combination is reported to prolong QT and lacks safety data for patients with arrhythmia or renal or hepatic impairment. So, robust clinical data are required to support the efficacy and safety of this combined therapy (Machiels et al., 2020). Recently an open-label and randomized phase-3 clinical trial of hydroxychloroquine and azithromycin for COVID-19 treatment in pregnant women has been withdrawn. In another UK-based, open label, multi arm, and randomized clinical trial, the routine use of azithromycin failed to display promising activities to shorten the recovery time or risk of hospitalization of COVID-19 infected individuals. The trial also reported inappropriate and excessive use of azithromycin during the COVID-19 pandemic which ultimately leads to antimicrobial resistance (Butler et al., 2021). A few other clinical studies also suggested the same inefficacy of azithromycin or combination of azithromycin with hydroxychloroquine in terms of virologic clearance and other poor clinical outcomes (Molina et al., 2020; Echeverría-Esnal et al., 2021). Besides, the administration of hydroxychloroquine and azithromycin in hospitalized patients has been linked to an increased risk of cardiac adverse events. More specifically, such a combination has been associated with an increased risk of QTc prolongation, ventricular arrhythmia, TdP (0.4%), atrial fibrillation, atrioventricular block, and cardiac failure (Echeverría-Esnal et al., 2021).

Interestingly, some very recent studies revealed that in combination therapy, hydroxychloroquine might cause cardiac toxicity not azithromycin (Echeverría-Esnal et al., 2021). In addition, in the only randomized-controlled clinical trial conducted to date in hospitalized patients, azithromycin monotherapy did not confer a higher risk of adverse effects like hydroxychloroquine or combination therapy with hydroxychloroquine (Arshad et al., 2020; Echeverría-Esnal et al., 2021). Thus, though a cautious risk-benefit ratio and follow up of ADRs should be monitored, from a safety perspective azithromycin could be being studied for its use against SARS-CoV-2 infection (Echeverría-Esnal et al., 2021).

### Lopinavir-Ritonavir Combination

Lopinavir-Ritonavir combination (Kaletra), approved by the FDA in 2000, was developed by Abbott Laboratories, USA to treat HIV patients. Both drugs are retroviral protease inhibitors but are given in combination to enhance Lopinavir exposure within the body. Lopinavir has a short half-life and so was co-formulated with ritonavir which can minimize extensive biotransformation of Lopinavir inhibiting the metabolizing enzyme cytochrome P450 (Chandwani and Shuter, 2008). With positive results from an *in-vitro* SARS-CoV inhibitory study, lopinavir/Ritonavir was first used in a clinical study with 152 patients during the 2003 SARS outbreak. This study reported weak antiviral activity of lopinavir-ritonavir and suggested further studies (Chu et al., 2004).

**FIGURE 4:**
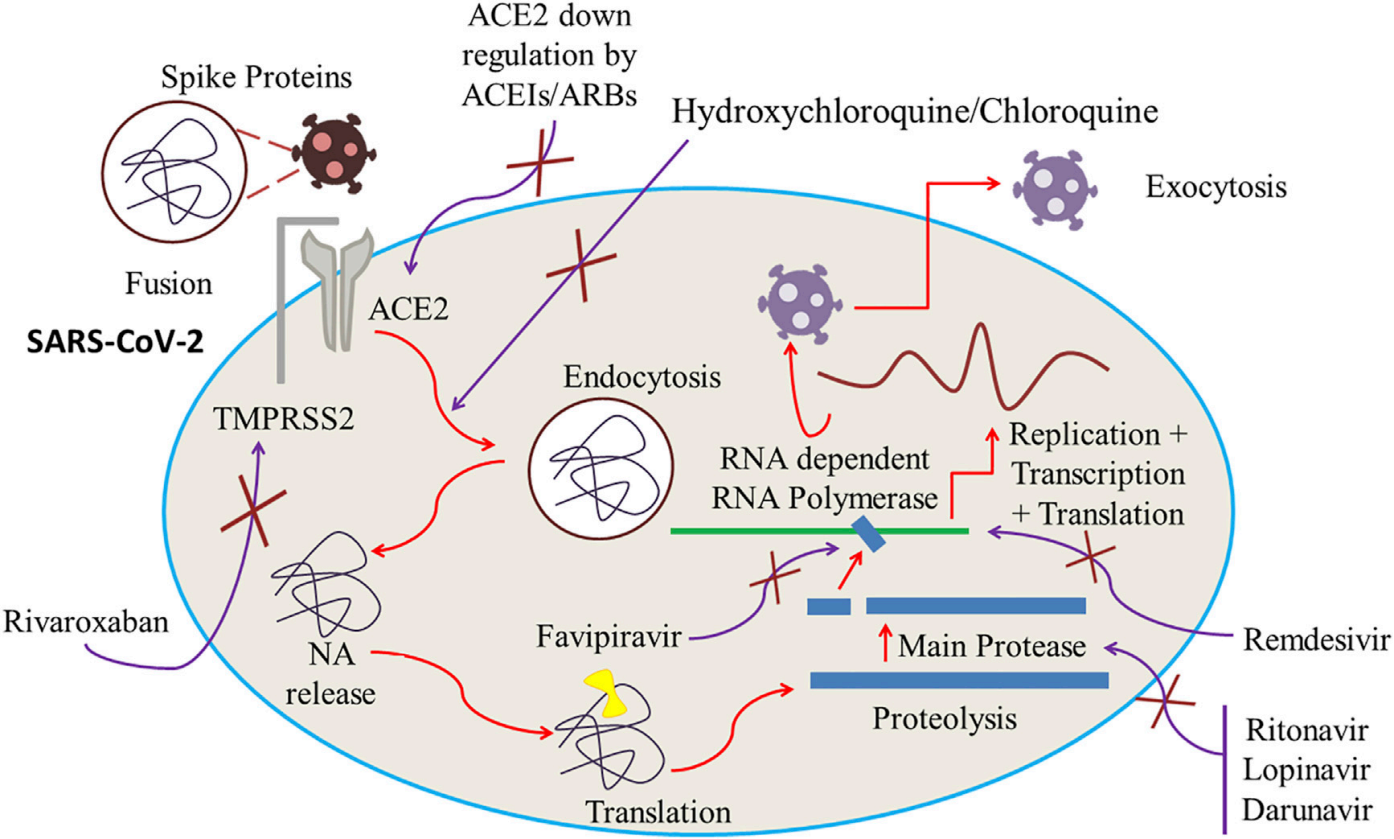
ACE2 downregulation by ACEIs/ARBs. Inhibition of RNA-dependent RNA Polymerase and main protease by, respectively, Favipiravir and Lopinavir/Ritonavir. Cessation of viral fusion onto the cell membrane and endocytosis by increasing the cell pH by Hydroxychloroquine/Chloroquine. Inhibition of transmembrane protease serine 2 (TMPRSS2) by rivaroxaban which can potentially block the viral entry of SARS-CoV-2 into the host by ceasing the fragmentation of spike protein into the S1 and S2 subunits.

Plausible effectiveness of lopinavir/ritonavir against SARS-Cov-2 might be due to the inhibition of viral cysteine protease. Several studies involving the use of lopinavir-ritonavir in SARS-Cov-2 patients have been conducted. These studies demonstrated positive results for old patients and pediatric patients and reported reduced hospital stay time with quick recovery. But in a recent study this combination showed adverse effects with no significant improvement in clinical recovery time and mortality rate (Singh et al., 2020). Anti-COVID-19 action of lopinavir–ritonavir combination is displayed in Figure 4.

In Korea, lopinavir–ritonavir therapy has been proposed for high-risk elderly COVID-19 patients at an early stage depending on a positive report from lopinavir/ritonavir application for treating COVID-19 pneumonia in a Korean patient (Lim et al., 2020). A retrospective case-series study involving 19 severe COVID-19 patients demonstrated 94.74% clinical improvement rate (Luo et al., 2021). A randomized controlled and open-label trial conducted by Cao et al. (2020) to investigate the specific efficacy of lopinavir/ritonavir administration showed no significant improvement of clinical recovery time compared to standard care (Cao et al., 2020). Though there is a lack of adequate clinical data to confirm the effectiveness of this combination, Chinese guidelines suggests the combination for critically ill elderly COVID-19 patients. Moreover, several case studies reported enhanced antiviral activity when lopinavir-ritonavir is administered with other drugs like ribavirin and interferon alfa (Khan et al., 2020). Triple combination of lopinavir/ritonavir, ribavirin, and interferon-β was compared with lopinavir/ritonavir in a recent multicenter, prospective, open-label, and randomized phase-2 trial. The study demonstrated significantly better clinical response and reduced median hospitalization (9 days against 14.5 days, *p* = 0.016) in the treatment group in comparison to the control group. In the study, 86 hospitalized patients who were early assigned (within a median of 5 days) to a triple combination of lopinavir/ritonavir (400/100 mg every 12 h), ribavirin (400 mg every 12 h), and three doses of 8 million international units of interferon-β 1b on alternate days for 14 days, had a significantly reduced median time of hospital stay [(7 days against 12 days, *p* = 0.0010) than the control group of 41 patients treated only with lopinavir/ritonavir for 14 days (Meini et al., 2020). Several clinical trials are ongoing with lopinavir-ritonavir alone and in combination with other drugs like Favipiravir to reach a conclusion regarding their effectiveness and safety for COVID-19 treatment. This combination is also being compared with other agents like hydroxychloroquine sulfate and darunavir/cobicistat in recent trials (Anonymous, 2020c). However, a retrospective case-series study raised concerns about the safety of lopinavir-ritonavir use in COVID-19 patients based on the possibility of causing serious adverse events even before reaching the presumed therapeutic concentration for COVID-19 treatment. Moreover, WHO reported the recommendation of Solidarity trial regarding immediate discontinuation of this combination in COVID-19 patients due to ineffectiveness and safety concerns on July 4, 2020 (Lepage et al., 2021). Again, a large retrospective, multicenter study involving 8,553 COVID-19 patients found no overall advantage of lopinavir-ritonavir administration in early COVID-19 patients (Lora-Tamayo et al., 2021). Moreover, based on data extracted from 17 different studies, the use of lopinavir-ritonavir was strongly discouraged by WHO due to their dubiousness on the rate of reducing mortality and mechanical ventilation along with the probability of increasing diarrhea (Lamontagne et al., 2020). Besides, the time of clinical improvement is also uncertain when lopinavir-ritonavir was used against COVID-19 infection (Lamontagne et al., 2020).

### Chloroquine

Chloroquine was first extracted from the cinchona plant by Bayer Laboratories in 1934 (Savarino et al., 2003; Singh et al., 2020). It has been used clinically for more than 50 years all over the world. Initially it was synthesized and applied as an anti-plasmodial drug, but it also can act as an extended spectrum antiviral agent (Savarino et al., 2003; Paton et al., 2011). Chloroquine and hydroxychloroquine are established drug moieties with great similarities in their structures (Rynes, 1997) though compared to chloroquine, hydroxychloroquine exhibits less toxicity due to the presence of a hydroxyl group (Wu et al., 2020). Chloroquine can restrain SARS-CoV-2 from entering into the host cell. It can also effectively inhibit the virus and host cell fusion by altering glycosylation of ACE2 receptor and its binding capacity with spike protein. There is also evidence of chloroquine’s efficacy during early phase of COVID-19 attack by down regulating expression of ACE2 and its activity (Savarino et al., 2006; Liu et al., 2020; Wang et al., 2020). The anti-COVID-19 action of chloroquine is displayed in Figure 4.

On the contrary, according to some research, chloroquine, evaluated against several viral infections, including human immunodeficiency virus (HIV), MERS-CoV, SARS-CoV, chikungunya, and dengue (Savarino et al., 2003; Keyaerts et al., 2009; Colson et al., 2020), failed to show any benevolent actions in animal models or clinical trials (Touret and de Lamballerie, 2020). According to research, during a randomized double blind placebo controlled trial, chloroquine could not deliver any promising activity against viral infections such as influenza virus (Paton et al., 2011).

Researchers in China recommended that during COVID-19 infections 500 mg chloroquine should be provided twice a day for ten days continuously (Jie et al., 2020; Smith et al., 2020). The China National Center for Biotechnology Development also documented chloroquine as one of the top three anti-COVID-19 drug candidates (Khan et al., 2020). Primary evidence from the Chinese authorities also stated that almost 100 COVID-19 affected patients experienced quicker recovery from fever, alleviated lung computed tomography (CT) images, and no pronounced side effects and recommended chloroquine in the COVID-19 treatment protocols (Gao J. et al., 2020; Jie et al., 2020). This made chloroquine one of the first line treatments throughout China and different countries, though some prevalent side effects, including cardiomyopathy and macular retinopathy, cannot be nullified (Cubero et al., 1993). Almost 10 clinical studies have been conducted to determine the efficacy and safety of chloroquine to ascertain improved therapeutic decision (Harrison, 2020; Smith et al., 2020). But some reports also stated that chloroquine in high doses (600 mg twice daily for 10 days or total dose of 12 g) can trigger cardiac arrest and thus should not be prescribed for COVID-19 infection (Borba et al., 2020). Despite being a safe choice, chloroquine, a drug with a narrow therapeutic index, can trigger retinopathy, methemoglobinemia, gastrointestinal anomalies, tinnitus, and cardiac conditions including arrythmias and cardiomyopathy (Frisk-Holmberg et al., 1983; Waldrop et al., 2020). As the safety and efficacy profiles of chloroquine and hydroxychloroquine is still under question, health practitioners should make a critical decision considering risk vs. benefit ratio (Juurlink, 2020).

Several clinical trials were conducted to evaluate the efficacy of chloroquine against COVID-19 infection. Up to April 2020, more or less 80 clinical studies had been conducted on chloroquine and/or hydroxychloroquine and even sometimes with other drugs, to assess the efficacy of these agents against the novel corona virus strain. A few trials revealed positive outcomes (Femere and Aronson, 2020). Moreover, several trials are underway to compare chloroquine with other drugs (like hydroxychloroquine or telemedicine) or to compare chloroquine combination drugs (chloroquine + losartan) with chloroquine alone (Anonymous, 2020d). Based on a trial conducted in 2020, the candidacy of chloroquine was proposed by alleviating disease symptoms and supporting virus negative conversion and shortening of the disease period (Gao et al., 2020). But after preliminary hype, the safety and efficacy profile of chloroquine or hydroxychloroquine has imposed a severe question mark on their repurposing use. Use of chloroquine/hydroxychloroquine can induce cardiac arrest and malignant arrhythmias. Another meta-analysis conducted on COVID-19 patients also revealed risk of QTc prolongation followed by discontinuation of chloroquine/hydroxychloroquine (Kashour et al., 2021). The increasing number of ADR incidences resulted from chloroquine/hydroxychloroquine revealed its failure in COVID-19 infection. The number of ADRs (from FAERS database) due to chloroquine and hydroxychloroquine is more than double in previous years (in 2020 *n* = 11,493 and 89,607 ADRs, in 2019 *n* = 5,131 and 37,559 ADRs and in 2018 *n* = 4,681 and 25,035 ADRs) which is possibly attributable to excessive use of these drugs against SARS-CoV-2 infection. Considering these incidences and major clinical trials, the USFDA suspended emergency use authorization for these drugs on June 15, 2020 (Bull-Otterson et al., 2020). Thus, with time, the once promising drug candidates chloroquine and hydroxychloroquine failed to exert their safety and efficacy profiles against COVID-19 infections and dropped out from the race of being repurposed.

### Hydroxychloroquine

Being an analog of chloroquine, hydroxychloroquine also shows immunomodulatory activities and is used to treat malaria, rheumatoid arthritis, and systemic lupus erythematosus (SLE) (Savarino et al., 2003; Wu et al., 2020). As a safer analogue of chloroquine, an FDA approved drug, hydroxychloroquine has been prescribed for the last 60 years and is also the most abundantly prescribed antimalarial drug (Shippey et al., 2018).

Despite not having an evidently elucidated mechanism of action of hydroxychloroquine against coronavirus, several mechanisms to irradiate its antiviral activity are already projected. Reports claim that hydroxychloroquine can effectively inhibit SARS-CoV-2 infection during *in vitro* evaluation (Colson et al., 2020; Liu et al., 2020; Zhou P. et al., 2020). Being a weakly basic drug, hydroxychloroquine increases the pH within cells and at the cellular membrane and consequently arrests the capability of the virus to fuse onto the cell membrane and invade the host cell. Other anticipated mechanisms of hydroxychloroquine include cessation of DNA and RNA synthesis along with immunomodulating and anti-inflammatory properties (Reilly, 2020). Hydroxychloroquine can also arrest glycosylation of viral proteins, virus assembly, new virus particle transport, virus release, and other processes to attain its antiviral activity (Vincent et al., 2005). The anti-COVID-19 action of hydroxychloroquine is displayed in Figure 4.

Hydroxychloroquine has been given priority in COVID-19 management in various countries due to its abundant availability and *in vitro* activity against COVID-19 (Khan et al., 2020). Clinical trials in mild, moderate, and severe cases ensure that hydroxychloroquine can eliminate viral nasopharyngeal carriage of COVID-19 within a week, though at present no report from Randomized Controlled Trials (RCTs) is available (Gautret et al., 2020; Khan et al., 2020). Despite not establishing a clear dosage regimen, a few USA experts recommend variable doses including 400 mg BID on day one and then continuation for 5 days, 400 mg BID on day one and then continuation of 200 mg BID for 4 days, and 600 mg BID on day one and then continuation of 400 mg daily on days 2–5 (Khan et al., 2020). The China International Exchange and Promotive Association for Medical and Health Care (CPAM) has given a guideline of hydroxychloroquine use (400 mg once daily) when chloroquine cannot be availed (Smith et al., 2020).

A recent study confirmed 8 out of 10 patients’ positive status for SARS-CoV-2 PCR on days 5–6 after treating with hydroxychloroquine and azithromycin (Molina et al., 2020). Another study which has not yet been peer reviewed reported clinical improvement in 80.6% cases (25 of 31 patients) when treated with hydroxychloroquine whereas there was a 54.8% (17 of 31 patients) improvement rate in control group who were treated with antivirals other than hydroxychloroquine (Chen Z. et al., 2020), though use of both chloroquine and hydroxychloroquine should be monitored strictly including checking of patients’ QT measurement. Thus, off-label use should be restricted (Giudicessi et al., 2020). A few patients should be tested for glucose-6-phosphate dehydrogenase deficiency as use of these drugs in compromised cases can result in hemolytic anemia (Vijayvargiya et al., 2020).

In contrast, based on clinical data from 76 different studies, WHO strongly recommended against the use of hydroxychloroquine and chloroquine in COVID-19 patients independent of their severity. In case of special considerations, if patients with a history of tachyarrythmias, ventricular tachycardia, prolonged QT interval, etc., need hydroxychloroquine or chloroquine, more precautions should be taken (Lamontagne et al., 2020). Data from 29 studies with 10,859 patients suggested the improbability of decreasing mortality and data from five studies indicated the unlikelihood of reduction of mechanical ventilation by hydroxychloroquine. Other studies also recommended the uncertainty of hydroxychloroquine’s effect on viral clearance, admission to hospital, and clinical improvement time (Lamontagne et al., 2020). A meta-analysis based on one randomized trial and 12 cohort studies that comprised of 19,573 hospitalized COVID-19 infected patients exerted no statistically significant relationship between hydroxychloroquine and mortality rate, while three cohort studies also reported no association of this drug with the abatement of mechanical ventilation and ICU administration requirements (Kashour et al., 2021). In addition, two more cohort studies also failed to display a statistically significant relationship between mechanical ventilation and ICU administration requirement in COVID-19 infection and hydroxychloroquine when co-administered with azithromycin (Kashour et al., 2021). Besides, according to Geleris et al., a study comprised of 1,446 hospitalized COVID-19 affected patients who received hydroxychloroquine showed no positive outcomes in lowering of death risk (Geleris et al., 2020). Another study conducted on 150 patients (hydroxychloroquine group = 75 and standard care group = 75) also failed to illustrate promising outcomes in terms of being COVID-19 negative (Tang W. et al., 2020). Considering all these factors, WHO decided to discontinue hydroxychloroquine as treatments arms against COVID-19 (Hossain and Rahman, 2020).

### Ivermectin

In 1974, isolation of a new strain of a soil-dwelling bacterium in the laboratories of the Kitasato Institute in Japan and subsequent transferal of the sample to the laboratories of Merck *&* Co., Inc. in USA, gave rise to the serendipitous discovery of an active anthelmintic substance. Thus, avermectins were identified and with piecemeal research works were subsequently transmuted into antiparasitic products such as ivermectin. After much scientific research, in 1981, ivermectin was presented commercially against endoparasitic nematode and ectoparasitic arthropods in livestock, though initially the target was to apply it in veterinary science and animal husbandry (Campbell, 2012).

**FIGURE 5:**
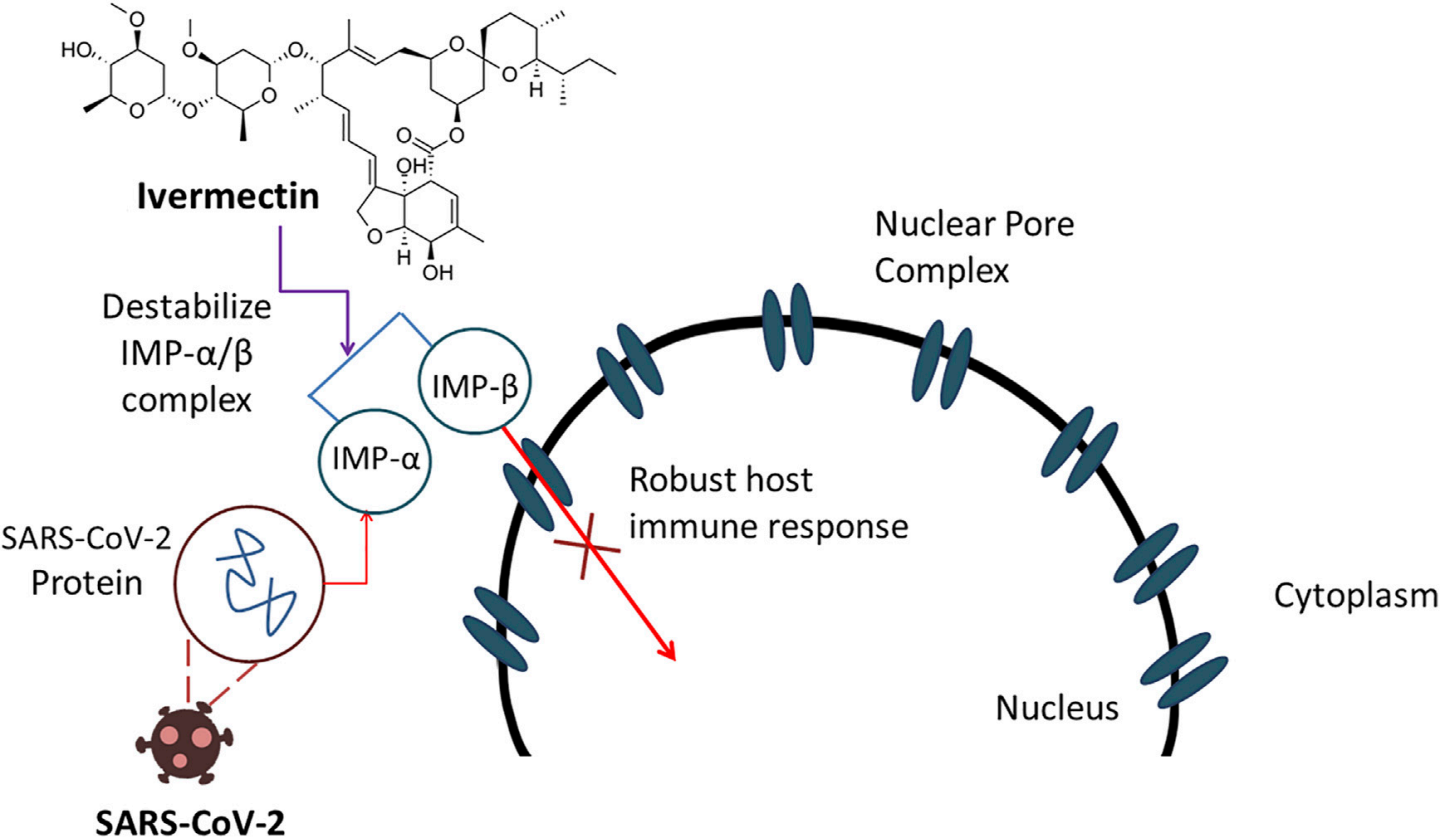
Ivermectin demonstrates anti-COVID-19 action by inhibiting nuclear transport resulted by destabilization of IMP-α/β complex which ultimately damages RNA viruses.

SARS-CoV-2, an RNA virus containing only a phospholipid envelope to preserve its genetic material with few protein inserted, does not show any protein capsid (Sigrist et al., 2020) which make them vulnerable against ivermectin (Cordova et al., 2003). Ivermectin can act upon this type of viruses by altering ionic balance between the internal and external environment by reentering water molecules, resulting in osmotic lysis (Rizzo, 2020). Thus, integrity and functionality of a viral membrane can be damaged seriously. Though the exact mechanism is not strongly elucidated, another report suggested that the probable anti-COVID-19 action of ivermectin is attributed by the inhibition of nuclear transport facilitated by the importin α/β1 heterodimer (Rizzo, 2020). This inhibitory impact can damage a notable sum of RNA viruses (Caly et al., 2012; Jans et al., 2019) including novel corona virus (Heidary and Gharebaghi, 2020). The anti-COVID-19 action of ivermectin is displayed in Figure 5.

According to a recent study, a single dose of ivermectin can reduce viral RNA of SARS-CoV-2 by 5000-fold in a Vero-hSLAM cell culture model at 48 h, which attracted researchers to consider it as a well-deserved drug candidate against COVID-19 attack, though antiviral concentration of ivermectin demands a large dose (Caly et al., 2020). On the other hand, the ability of this drug to cross the blood brain barrier and affect GABAergic transmission at high doses makes its safety profile questionable (Hossen et al., 2020). A safe dose of ivermectin is considered as ≤ 200 μg/kg and overdose of this drug can cause severe adverse effects such as ataxia, psychosis, depression, seizure, and confusion (Gritti et al., 2020). Thus, dose can be a huge concern in ivermectin therapy and challenge the treatment protocol during COVID-19 infection.

Several clinical trials are being conducted with ivermectin alone or in combination to conclude about the efficacy and safety of the drug in COVID-19 patients. A recent double-blind randomized clinical trial confirmed early clinical improvement of COVID-19 patients with ivermectin and doxycycline combination compared to standard care (Hashim et al., 2020). Ivermectin was also subjected to another clinical trial among 72 hospitalized Bangladeshi SARS-CoV-2 infected adults as a randomized, placebo controlled, and double-blind testing to ensure the safety profile along with the ability of COVID-19 clearance. During this trial, patient pools were divided into three subgroups, where each group individually received either 12 mg ivermectin monotherapy once daily for 5 days or combination therapy comprised of 12 mg ivermectin and 200 mg doxycycline on day 1 followed by 100 mg of doxycycline every 12 h for next 4 days and the last subgroup was assigned as a placebo control group. Virological clearance reported from these three groups was respectively 9.7, 11.5, and 12.7 days and thus this study demonstrated efficacy of ivermectin therapy in COVID-19 cases without any severe adverse effects (Ahmed et al., 2021). In another study, ivermectin was also subjected to RT-PCR proven COVID-19 affected patient in two different doses comprised of 6 and 12 mg in every 84 h pattern for two weeks and compared with control (lopinavir/ritonavir daily for 2 weeks) group. In this trial, mean days to negative were respectively 6.0, 4.65, and 9.15 days which demonstrated the candidacy of Ivermectin 12 mg in the clinical management of COVID-19 infection (Babalola et al., 2021). Besides, another clinical trial conducted on 600 participants showed significant abetment in RT-PCR conversion days along with mortality rate. This study recommended early use of ivermectin in COVID-19 infection as prophylaxis and to improve cytokines storm (Elgazzar et al., 2020). Moreover, in accordance with the Iranian Registry of Clinical Trials website (registration ID IRCT20200408046987N1), ivermectin is also a notable adjunct to reduce mortality rate, low O_2_ duration, and hospitalization days among adult COVID-19 infected adults with a broad margin of safety and high therapeutic impact (Niaee et al., 2020).

On the contrary, one study conducted in Pakistan that comprised of 50 patients in total (equally divided into case group and control group where both groups received symptomatic treatments but ivermectin was only prescribed to case group) failed to demonstrate any statistically significant outcomes against COVID-19 infection and suggested that symptomatic treatments can be equally effective with or without ivermectin (Chachar et al., 2020). In another study, conducted among 62 mild to moderate SARS-CoV-2 infected patients, ivermectin again failed to demonstrate significant beneficial effects. In that study, total recovery time recorded for ivermectin group (ivermectin 200 μg/kg + symptomatic treatment) and control group (only symptomatic treatment) was 10.09 ± 3.236 and 11.50 ± 5.32 respectively which discarded the standing of ivermectin therapy in mild to moderate SARS-CoV-2 infection (Podder et al., 2020). Again, WHO recommended against the use of ivermectin in COVID-19 patients except for clinical trials due to the unpredictable association with ivermectin and important clinical outcomes like mortality rate, mechanical ventilation, hospital admission, and duration of hospitalization based on data from 31 different studies (Lamontagne et al., 2020).

### Favipiravir

Favipiravir was discovered many years ago during the primary *in vitro* experimentation for anti-influenza activity by chemical remodeling of a pyrazine analogue at Research Laboratories of Toyama Chemical Co., Ltd. In essence, it blocks the influenza viral RNA polymerase inhibiting viral transcription process (Furuta et al., 2017; Khambholja and Asudani, 2020). Favipiravir, one of the pronounced antiviral agents, has been tested against several SARS-CoV-2 envelope proteins. Favipiravir, a pyrazine carboxamide derivative molecule, acts as an antiviral molecule which is basically a prodrug against RNA viruses and desired antiviral property is attenuated by the competitive nature of favipiravir with purine nucleoside rather than pyrimidine nucleosides. Madin Darby Canine Kidney (MDCK) cell treated with favipiravir during *in vitro* testing provided favipiravir ribofuranosyl-5′-triphosphate (favipiravir-RTP), favipiravir ribofuranose (favipiravir-R) and favipiravir ribofuranosyl-5′-monophosphate (favipiravir-RMP) in HPLC analysis as metabolites among which chemically synthesized favipiravir-RTP demonstrated to the ability to obstruct the viral RNA polymerase activity in concentrations ranging from nanomolar to micromolar. This test reflects the projection that favipiravir, which is a prodrug, can emit an antiviral property when it is intracellularly phosphoribosylated to be an active form, favipiravir-RTP, to inhibit RNA polymerase. When a single molecule of Favipiravir-RTP is proficiently incorporated into a nascent RNA strand, a partial cessation of extension in viral RNA is detected and when double incorporation takes place, total inhibition results in (Furuta et al., 2017). The anti-COVID-19 action of favipiravir has been displayed in Figure 4.

Favipiravir has demonstrated prominent therapeutic impacts in COVID-19 treatment by arresting infection progression and viral removal and research has also stated that, due to mild adverse effects, the treatment duration of favipiravir can be prolonged as per requirements (Cai et al., 2020). In accordance with the official at the Chinese Ministry of Science and Technology along with the Guardian news report, favipiravir has exhibited auspicious clinical trials outcomes in Shenzhen and Wuhan in a study comprised of 340 patients (McCurry, 2020). The Turkish Ministry of Health has also focused on favipiravir in intensive care cases to decrease infection periods ranging from 11–12–4 days (Al-Monitor News, 2020). A recent nonrandomized, controlled, open-label trial reported efficacy of favipiravir in COVID-19 affected patients (1,600 mg twice in day 1 followed by 600 mg twice in day 2–14) compared to lopinavir-ritonavir combination including improved chest imaging reports in favipiravir group (91.43%) than lopinavir-ritonavir group (62.22%) on day 14 of medication (Cai et al., 2020). On the contrary, favipiravir, a teratogenic agent, cannot be prescribed in pregnancy cases (Delang et al., 2018) and thus it demands more vigorous clinical trials from concerned regulatory bodies controlling medical care in each country to get the approval of widespread utilization (Khan et al., 2020).

At present, a few clinical trials of favipiravir are ongoing throughout several countries, including China and Japan, to reestablish the therapeutic profile of favipiravir against COVID-19 infection (Wu et al., 2020). A randomized control trial (ChiCTR200030254) has displayed that infected individuals treated with favipiravir experienced a faster recovery rate (71.43%) compared to treated individuals with umifenovir (55.86%), whereas fever duration and cough relief time are also alleviated prominently (Chen, C. et al., 2020). Up to mid-April, 2020, there were two clinical trials in Japan and eight in China under way in order to evaluate the efficacy of favipiravir against novel coronavirus. These clinical studies included non-randomized and randomized controlled trials assessing the safety and efficacy of favipiravir alone (ChiCTR2000030113, JPRN-jRCTs031190226, JPRNjRCTs041190120) or in conjunction with interferon-α (C h i C T R 2 0 0 0 0 2 9 6 0 0), baloxavir marboxil (ChiCTR2000029544, ChiCTR2000029548), tocilizumab (ChiCTR2000030894, NCT04310228), or chloroquine phosphate (ChiCTR2000030987, NCT04319900) (Wu et al., 2020). Currently, several trials are ongoing in Italy, Saudi Arabia, Turkey, the United States, Iran, Russia, Germany, Mexico, Indonesia, Kuwait, Egypt, Bangladesh, Nepal, the United Kingdom, Hungary, and Thailand to evaluate the performance, safety, and efficacy of favipiravir alone or in combination with other agents and in comparison to other agents for mild, moderate, and severe COVID-19 treatment (Anonymous, 2020e). Five ongoing clinical trials are predicted to provide promising results for the use of favipiravir in COVID-19 treatment. This drug can be considered as a relatively safer treatment option for COVID-19 patients than other agents except in pregnancy due to teratogenicity (Maslub et al., 2021). A recently published preprint of a retrospective observational case control study (between March and September 2020 in Turkey) suggested the early use of favipiravir in COVID-19 treatment for better clinical outcomes and viral load reduction (Uçan et al., 2021). Three case-reports, one involving two elderly COVID-19 patients, another with a case of COVID-19 pneumonia, and the third with a critically ill patient, supported the use of favipiravir for beneficial effect in COVID-19 treatment. A randomized multicenter study including 96 COVID-19 patients performed between April and August 2020 reported reduced hospital stay and decreased need for mechanical ventilation with favipiravir than with Chloroquine (Dabbous et al., 2021). Another randomized, open-label, comparative, multicenter phase-3 clinical trial from May 14 to July 3, 2020 with 150 patients suggested the benefit of favipiravir in mild to moderate COVID-19 based on the median time of viral shedding cessation [5 days (95% CI: 4 days, 7 days) vs. 7 days (95% CI: 5 days, 8 days), *p* = 0.129] and clinical cure (3 days (95% CI: 3 days, 4 days) vs. 5 days (95% CI: 4 days, 6 days), *p* = 0.030) compared to control (Udwadia et al., 2021). On the other hand, Medisetty et al. (2021) expressed concern about the conclusion made by Udwadia et al. (2021) and proposed the need for well-designed randomized, placebo controlled clinical trials for clearer evidence before using favipiravir in mild to moderate COVID-19 cases (Medisetty et al., 2021; Udwadia et al., 2021).

However, several studies demonstrated mixed results of efficacy and a complex pharmacokinetic profile of the prodrug, favipiravir, leading to dose determination challenges for COVID-19 treatment. Due to pharmacokinetic complexities and a lack of correlation between favipiravir concentration and viral load reduction, assessment of the active triphosphate form concentration in cell and tissue is required to understand its pharmacokinetic/pharmacodynamic profile. Thus, the challenges to achieve effective drug concentrations should be considered if using favipiravir to treat COVID-19 (Ison and Scheetz, 2021).

### Rivaroxaban

In today’s market, there are four clinically approved direct FXa inhibitors to be used as anticoagulants: rivaroxaban (approved in 2011; Xarelto) (Mueck et al., 2008), apixaban (2012; Eliquis) (Byon et al., 2019), edoxaban (2015; Savaysa) (Bounameaux and Camm, 2014), and betrixaban (2017; Bevyxxa) (Garland et al., 2018). In 2008, Bayer Healthcare was given approval for clinical use of rivaroxaban under the brand name Xarelto as an orally administered direct factor Xa inhibitor in order to prevent venous thromboembolism (VTE) after elective hip or knee replacement surgery (Perzborn et al., 2011). An early validation research on naturally occurring inhibitors for factor Xa resulting in the development of synthetic and orally selective factor Xa inhibitors in 1998 recommended rivaroxaban’s potency and specificity along with good oral bioavailability. The preclinical profiles of rivaroxaban also ensure high oral bioavailability, dose-dependent pharmacokinetics, a faster onset/offset of action, negligible drug-drug or drug-food interactions, and ascertains rivaroxaban as prolific drug candidate which does not demand routine coagulation monitoring (Perzborn et al., 2011).

The spike protein of SARS-CoV-2 is comprised of a couple of vital subunits: the S1 subunit containing the host receptor binding domain and the S2 subunit, accountable for the fusion of virus and host cell membrane. The S1 subunit binds to its receptor ACE2 (angiotensin-converting enzyme 2) on the host cell while a proteolytic activation promoted by the host protease including transmembrane protease serine 2 (TMPRSS2), furin, and liposomal cathepsins results in the breakdown of the linkage between S1 and S2 subunits to ease the fusion (Belouzard et al., 2009; Shang et al., 2020). A few other host proteases like Human Factor Xa (FXa) also possess noteworthy viral entry and fusion regulation property (Belouzard et al., 2009; Shang et al., 2020). It is also reported that those FXa inhibitors can potentially block the viral entry of SARS-CoV-2 into the host by ceasing the fragmentation of spike protein into the S1 and S2 subunits. Thus, the concentration dependent inhibitors can obstruct the SARS-CoV-2 plaque formation in Vero E6 cells and thwart the entry of corona virus (Al-Horani, 2020). Additionally, a few recent studies have suggested that FXa inhibitors can potentially inhibit TMPRSS2 and act against a wide spectrum of RNA and DNA viruses by ceasing the viral entry phase (Du et al., 2007; Kim et al., 2020). Therefore, FXa inhibitors can be a potential agent against COVID-19 attack (Al-Horani, 2020). The anti-COVID-19 action of rivaroxaban is displayed in Figure 4.

Direct FXa inhibitors possess significant anticoagulant, anti-inflammatory, and antiviral properties which make them potential candidates in COVID-19 management (Al-Horani, 2020). At present, more than 10 clinical studies are being carried out to investigate the role of these agents against novel corona virus where dosage regimen is kept similar to their conventional use given to thrombotic patients after considering renal function (Al-Horani, 2020). A few trials suggested usage of these agents, such as rivaroxaban for 21–30 days, though more concrete reports are still needed to ensure the desired benefits (Al-Horani, 2020). Another study conducted on 183 hospitalized patients in China documented that activation of the coagulation system can play a detrimental role in COVID-19 management (Tang N. et al., 2020). According to that research, the degree of activation of a coagulation system such as increased D-dimer concentrations during admission is much higher in non-survival cases. Overall, 21 (11%) of 183 patients died; 15 (71%) of the 21 non-survivors and only one (1%) of the 162 survivors met criteria for disseminated intravascular coagulation during their hospital stay. In a case series in China, elevated D-dimer concentrations during hospital admission (>1 μg/ml) was correlated with higher chances of death rate which is 18 times greater than normal individual (Zhou F. et al., 2020). Thus, the conducted study warned about a poor outcome in COVID-19 management if standard supportive therapy is absent (Zhou F. et al., 2020) and strongly recommended inclusion of thromboprophylaxis in corona virus treatment (Spyropoulos et al., 2020).

A few clinical trials are ongoing in the United States, Germany, Brazil, and the United Kingdom to evaluate the safety and efficacy of rivaroxaban alone or in combination with other drugs for moderate to severe COVID-19 treatment or for minimizing risk of major venous and arterial thrombotic events, hospitalization, and death in symptomatic COVID-19 infection (Anonymous, 2020f). Among them PREVENT-HD, a double-blind, placebo-controlled, pragmatic, event-driven phase 3 trial, started in August 2020 and was expected to enroll 4,000 participants to evaluate the efficacy and safety of rivaroxaban in COVID-19 outpatients with possible risk of thrombotic events, hospitalization, and death (Capell et al., 2021). A descriptive and analytical study with 50 COVID-19 patients (affected between April and May 2020) presenting pneumonia suggested the use of rivaroxaban for thromboprophylaxis (Carpio-Orantes et al., 2021).

### ACEIs/ARBs

For more than 30 years, ACEIs, the first medication to target the Renin Angiotensin Aldosterone System (RAAS), have been used to treat a variety of indications associated with hypertension, cardiovascular diseases, and renal diseases (Yehualashet and Belachew, 2020). ARBs can also show efficacy in hypertensive disorders. Not only do ACEIs and ARBs reduce blood pressure, but they also demonstrate distinctive cardio protective efficacy (Maione et al., 2011).

SARS-CoV and other SARS-related coronaviruses (SARSr-CoV) can directly interact with ACE2 to penetrate to the target site. Studies found that ACE2 is strongly pronounced in the mouth and tongue, promoting viral entrance in the host. ACE2 is found on type I and II alveolar epithelial cells of lower lungs. After invasion, the penetration of SARS-CoV-2 begins with the adherence to ACE2 on the alveolar surface of the spike glycoprotein distributed on the viral envelope. Clathrin-dependent endocytosis of the SARS-CoV-2 and ACE2 complexes modulates the adhesion of SARSCoV-2 to ACE2, prompting cell membrane fusion (Yehualashet and Belachew, 2020). SARS-CoV-2 uses the endogenous transcriptional machinery of alveolar tissues to replicate and spread throughout the entire lung once it enters the cells (Perico et al., 2020). ACE2 can aid SARS-CoV-2 S-mediated cell entry and thus establish it as an operational receptor for COVID-19. In the pathogenesis of COVID-19, angiotensin-converting enzyme inhibitors (ACEIs) and angiotensin receptor blockers (ARBs) have a distinct mechanism. ACEIs in clinical application cannot directly interfere with the activity of ACE2 (Brojakowska et al., 2020) and the intestinal messenger RNA concentration of ACE2 may be enhanced with ACEI but not reported with ARBs. SARS-CoV-2 abates the expression of ACE2, which resulted in reduction of the protective actions of ACE2 on various organs (Wysocki et al., 2020). The entrance is amplified by ACEIs more than twice compared to ARBs. This will certainly not infer the drawbacks or advantages of such treatments with patients displaying several variables such as age, hypertension, and the result of unspecified comorbidities on the outcome of the COVID-19 pandemic (Guo J. et al., 2020; Leclézio et al., 2020). By stabilizing ACE2-AT1R interaction and suppressing viral protein and ACE2 interaction and invasion, ACEIs or ARBs can arrest COVID-19 viral entrance. In the presence of stabilized ACE2-AT1R complexes, the interaction between the viral protein and ACE2 can be reduced in a significant manner (Leclézio et al., 2020). According to Yang et al. (2020), elevated cases of hypertension were found in serious COVID-19 patients though it is not concluded that the severity of this infection is associated with hypertension. Additionally, no strong data on the use of ACEIs and ARBs for COVID-19 infection has been published yet (Yang et al., 2020). According to Guo T. et al. (2020), during the evaluation of cardiovascular effects of COVID-19 infection, the use of ACEIs/ARBs failed to demonstrate any correlation with mortality case (Guo T. et al., 2020). However, previous studies on animal models have shown that ACEIs and ARBs can upsurge the action of ACE2. Thus, it is projected that ACEIs and ARBs, by improving the activity of ACE2, can increase COVID-19 infectivity. Some researchers have suggested that the use of ACEIs/ARBs may be effective in preventing COVID-19 infection (Li et al., 2017). The anti-COVID-19 action of ACEIs/ARBs is displayed in Figure 4.

There are several study reports on the use of ACEIs/ARB in COVID-19 which include mainly hospitalized patients. One study involving 1,128 hypertensive patients, 188 receiving ACEIs/ARB, showed in-hospital use of these drugs can reduce mortality risk (Zhang et al., 2020). In another larger study with 8,910 patients, 770 receiving ACEI and 556 receiving ARBs, decreased in-hospital mortality rate was shown for ACEI but not for ARBs (Mehra et al., 2020). A cohort study with 8.3 million people concluded that ACEI/ARBs use is associated with decreased risk of COVID-19 for in-hospital patients. But initial reports from New York showed no evidence of a beneficial outcome for ACEI/ARB use (Hippisley-Cox et al., 2020).

However, use of ACEIs/ARB in COVID-19 is controversial. Several studies on animals have suggested these to be beneficial while some studies have shown the risk associated with their use in COVID-19 patients. More studies are required to draw a conclusion about the benefit/risk ratio (Onweni et al., 2020). Currently a randomized clinical trial in Denmark is underway to investigate the effects of continuation and discontinuation of ACEIs/ARB in COVID-19 patients and another multicenter, randomized trial with 208 participants is being conducted to test the positive outcome of stopping ACEI/ARBs in symptomatic SARS-CoV-2 patients (NCT04351581, 2020; NCT04353596, 2020). Other trials with ACEI/ARBs are being conducted in Ireland, the United States, France, Canada, Saudi Arabia, Ukraine, Brazil, Spain, and Italy (Anonymous, 2020g).

A recent preprint of a retrospective cohort study suggested higher survival rates for in-hospital high-risk COVID-19 patients with the use of ARBs than ACEIs (de Abajo et al., 2021). Another retrospective cohort study in Wuhan suggested the beneficial role of ACEI/ARBs over calcium channel blockers in COVID-19 patients with hypertension (Liu et al., 2021). A recent study involving 53 COVID-19 patients hospitalized from February 8, 2020 to February 24, 2020 concluded that discontinuation of ACEIs/ARBs in hypertensive COVID-19 positive patients increases length of hospital stay (Tian et al., 2021). A meta -analyses of the existing observational cohort studies (including more than 22,000 COVID-19 patients) on the use of ACEIs/ARBs in COVID-19 found that ACEIs/ARBs was not associated with increased mortality, disease severity, or hospital stay in COVID-19 patients, but rather showed association with reduced mortality, severity, and hospitalization (Zhang et al., 2021). On the contrary, a retrospective cohort study with 182 hypertensive COVID-19 patients found no significant association between severity of COVID-19 or mortality and the use of ACEIs/ARBs (Abdullah et al., 2021). Another preprint of a Meta-Regression Analysis (consisting of 53 cross-sectional, case-control, and cohort studies published before January 18th, 2021 involving 112,468 patients) showed no effect on mortality and severity in COVID-19 patients using ACEIs/ARBs (Singh et al., 2021).

### Protease Inhibitors

The coronavirus glycoproteins allow viral entrance into target cells by attaching to receptors and accelerating viral and host cell membrane fusion. Nevertheless, to acquire a fusion-active form, glycoproteins are synthesized as inactive precursors and rely on activation by host cell proteases (Zhou et al., 2015). As a result, for acquiring broad-spectrum antiviral action, the respective enzymes can be considered as possible targets, and an effective guiding principle for the production of broad-spectrum antiviral medications will be the development of protease inhibitors (Zhou et al., 2011). Protease is also a crucial replication enzyme in the case of SARS-CoV-2 and is beneficial for the development of wide-spectrum inhibitors. The protease is responsible for converting it into functional proteins and its activity is induced by residues binding to particular points on the protease known as active sites where inhibitors can block the aforementioned activity (Wang et al., 2016; Mothay and Ramesh, 2020). When an inhibitor binds to an active site, it prevents substrates from binding, resulting in complete inhibition of the protease's action. Thus, a groundbreaking step to battle this pandemic could be the development of an inhibitor for COVID-19's protease and with this hope several protease inhibitors are now under clinical trials including PF-07304,814, EIDD-2801, and AT- 527 (Mothay and Ramesh, 2020). Therefore, despite not having any completed clinical studies, drugs of this class are included in this review work, so that they can get much deserved priority on being repurposed in COVID-19 infection.

PF-07304,814 is a notable phosphate prodrug which is converted into PF-00835321 and a strong inhibitor of 3CLpro that shows promising antiviral efficacy against SARS-CoV-2 as a monotherapy or combined therapy with remdesivir (Boras et al., 2020). A phase 1 clinical trial sponsored by Pfizer is in recruiting status to assess the safety, tolerability, and pharmacokinetic parameters associated with single and multiple doses of PF-07304814 in Hospitalized SARS-CoV-2 infected patients. In this triple masking, placebo controlled trial, 72 participants were enrolled where both PF-07304814 and placebo were introduced intravenously. Researchers are estimating that the completion of the clinical trial and outcomes can be available in July 5, 2021 (NCT04535167, 2020) and to our best knowledge, along with this uncompleted clinical trial, there is another clinical trial on PF-07304814 (also sponsored by Pfizer) which has been completed and registered in ClinicalTrails.gov till 8th April, 2020 to evaluate the candidacy of PF-07304814 against SARS-CoV-2 (NCT04627532, 2020).

EIDD-2801, also known as MK-4482 or molnupiravir, is a protease inhibitor and prodrug of ribonucleoside analog β-D-N^4^-hydroxycytidine (NHC) which showed efficacy against SARS-CoV and MARS-CoV in mice model. Previous records have generated enough interest amongst researchers to launch clinical trials of EIDD-2801 to evaluate its efficacy to alleviate SARS-CoV-2 infection (Wahl et al., 2021). Like PF-07304814, very few completed clinical trials (NCT04392219 and NCT04405570) on EIDD-2801 have been completed while only four clinical trials are enlisted in ClinicalTrials.gov till 8th April, 2020 (NCT04392219, 2020; NCT04405570, 2020).

AT-527, a guanosine nucleotide analog, is an orally administered double prodrug that can selectively obstruct the viral RNA-dependent RNA polymerase analog (RdRp). During *in vitro* evaluation against several 76 human coronaviruses, including SARS-CoV-2, AT-511, the free base of AT-527, has showed promising antiviral activity where the other active metabolite is AT-9010. The estimated concentration of the active metabolites in pulmonary tissues after oral dosing indicates that AT-527 can be an excellent therapeutic choice against SARS-CoV-2 infection (Good et al., 2021). There are only two clinical trials of this therapy against COVID-19 enlisted in ClinicalTrials.gov which are still in recruiting status and the estimated completion period is June, 2021 (NCT04396106, 2020; NCT04709835, 2020).

## Conclusion

COVID-19 is one of the biggest pandemic situations in the past hundred years. In the current scenario, drug repositioning can be a feasible option along with available vaccines. Several *in silico, in vitro,* and *in vivo* studies along with clinical trials, case series, retrospective studies, cohort studies, and meta-analyses have been conducted to establish the safety and efficacy profile, mechanism of action, pharmacokinetics, pharmacodynamics, and future aspects of repurposing drugs against COVID-19. Although several drugs have shown their effectiveness against SARS-CoV-2 infection in some notable studies and been used in many countries, safety and efficacy profiles of those drugs are yet to be well established in large scale clinical trials. Based on current study findings, some drugs clearly failed to be effective in the treatment of COVID-19. As a consequence, WHO has recommended against the use of such types of drugs (namely chloroquine, hydroxychloroquine, remdesivir, and lopinavir/ritonavir) in the treatment of COVID-19 infection. Thus, establishing complete safety and efficacy profiles of the trial drugs is still a desperate call and collaborative efforts throughout the world are needed to achieve this aim.
